# Dissolution of Arsenic Minerals Mediated by Dissimilatory Arsenate Reducing Bacteria: Estimation of the Physiological Potential for Arsenic Mobilization

**DOI:** 10.1155/2014/841892

**Published:** 2014-03-02

**Authors:** Drewniak Lukasz, Rajpert Liwia, Mantur Aleksandra, Sklodowska Aleksandra

**Affiliations:** Laboratory of Environmental Pollution Analysis, Faculty of Biology, University of Warsaw, Miecznikowa 1, 02-096 Warsaw, Poland

## Abstract

The aim of this study was characterization of the isolated dissimilatory arsenate reducing bacteria in the context of their potential for arsenic removal from primary arsenic minerals through reductive dissolution. Four strains, *Shewanella* sp. OM1, *Pseudomonas* sp. OM2, *Aeromonas* sp. OM4, and *Serratia* sp. OM17, capable of anaerobic growth with As (V) reduction, were isolated from microbial mats from an ancient gold mine. All of the isolated strains: (i) produced siderophores that promote dissolution of minerals, (ii) were resistant to dissolved arsenic compounds, (iii) were able to use the dissolved arsenates as the terminal electron acceptor, and (iii) were able to use copper minerals containing arsenic minerals (e.g., enargite) as a respiratory substrate. Based on the results obtained in this study, we postulate that arsenic can be released from some As-bearing polymetallic minerals (such as copper ore concentrates or middlings) under reductive conditions by dissimilatory arsenate reducers in indirect processes.

## 1. Introduction

Arsenic is considered to be one of the most hazardous elements, wherein its toxicity is revealed only when it is present in aqueous or gaseous form. Most of the arsenic-bearing minerals, such as arsenides and sulfarsenides, are considered nontoxic because they are highly insoluble. Problems arise when these primary minerals break down and enter into solution or form more soluble species such as oxides [1]. One of such cases, a common phenomenon in metallurgy industry, is arsenic release from ores and deposits into the environment through mining and smelting operations.

The most abundant and dominant arsenic ore minerals are As-sulfides, including arsenopyrite (FeAsS), realgar (As_4_S_4_), and orpiment (As_2_S_3_) [2]. Arsenic minerals are also found as impurities (as minor ores) in the ores of other metals such as gold or copper. Arsenopyrite is very often found in gold ores, while enargite (Cu_3_AsS_4_) and tennantite ((Cu,Fe)_12_As_4_S_13_) are the most common arsenic-bearing minerals associated with copper sulfide ore bodies [3]. Moreover, the fine fraction of ash produced by smelting of ore concentrates causes the airborne dispersion of arsenic, thus contaminating soil and streams over a wide area. A high arsenic content causes problems in the smelting and further extraction of metals, resulting in a reduction in the quality of the final product [3–5]. Environmental problems are also connected with the arsenic-bearing flotation tailings and mine waters. Arsenic present in these “reservoirs” can be transformed by the microbial activity and this may contribute to the further dissemination of arsenic contamination.

The most common microbial arsenic mobilization processes involve oxidative dissolution of minerals through oxidation of iron, sulfur, or arsenic [6]. Iron oxidizers can promote oxidative dissolution of arsenic minerals through direct or indirect mechanisms, which results in the production of toxic arsenious (H_3_AsO_3_) and arsenic acids (H_3_AsO_4_) [7]. In turn, oxidation of arsenic-bearing sulfide minerals causes not only the release of arsenic but also the acidification of waters and their enrichment in sulfate anions and the accompanying heavy metals [8]. Arsenic minerals dissolution can be mediated also at circumneutral pH, but mainly by sulfur- and arsenite-oxidizing microbes. It has been documented that the release of pyrite-bound arsenic may be caused by (i) oxidation of sulfide in the pyrite lattice [9] or (ii) direct oxidation of arsenic from mineral structure (e.g., arsenopyrite) [10]. The release of arsenic from As-bearing minerals may be also mediated by microbial reductive dissolution. Under reducing conditions, microorganisms can use arsenic compounds as terminal electron acceptors in arsenic respiration [11, 12]. However, this process is thought to be dominant for arsenic displacement from secondary minerals, for example, arsenate adsorbed on scorodite [13] and ferrihydrite [14]. There is no data about dissimilatory reduction of primary arsenic minerals, such as enargite, fangite, or luzonite, in which arsenic occurs as (sulf) arsenates. Thus, the question is whether the microorganisms are capable of removing arsenic from primary arsenic minerals through reductive dissolution. The answer to this question will help to complete the missing knowledge about microbial dissolution of arsenic-bearing minerals and will help to estimate the chance of the potential use of dissimilatory reducers in biomining and bioremediation. The possibility of selective removal of As(III) compounds (whose solubility is higher than As(V) and the level of As(III) sorption on the surface of secondary minerals is much lower and unstable) will be particularly important in industrial processing of As-bearing polymetallic minerals (such as copper ore concentrates or middlings (intermediate during ore processing)), where the presence of arsenic causes many technological and environmental problems.

The aim of this study was characterization of the isolated dissimilatory arsenate reducing bacteria in the context of their potential for arsenic removal from primary arsenic minerals through reductive dissolution. We demonstrated that the isolated strains (i) produce metabolites that promote dissolution of minerals, (ii) are resistant to dissolved arsenic compounds, (iii) are able to use the dissolved arsenates as terminal electron acceptor, and (iv) are able to remove arsenic under anaerobic conditions from As-containing copper ore concentrates and middlings, in which copper minerals were used as the sole terminal electron acceptor.

## 2. Materials and Methods

### 2.1. Isolation of Arsenate Dissimilatory Reducing Bacteria, Media and Growth Conditions

Microbial mats samples were collected from an ancient gold mine located in Zloty Stok, Lower Silesia, SW Poland, and were used as the inoculum for isolation of dissimilatory arsenate reducers. Microbial mats and the mine waters are characterized by high arsenic content (~5000–6800 mg/L for mats and ~3000–7000 *μ*g/L for mine waters), slightly alkaline pH (~7, 4–8.0), and stable temperature of 10–12°C throughout the year. 5 mL of microbial mats samples was added to the modified mineral salt medium (MSM) [10] (final volume, 100 mL) containing 2.5 mM sodium arsenate and 5 mM sodium lactate. Cultures were carried out in serum bottles with CO_2_ : N_2_ (in a ratio 20 : 80) injected into the headspace. These bottles were sealed and corked with silicon stoppers secured by aluminium crimp seals. Sampling was performed under CO_2_ : N_2_ (in a ratio 20 : 80) atmosphere in anaerobic glove box (Sigma-Aldrich). Cultures were incubated for 7 days at 22°C and then were subcultured twice for 7 days each. The cultures were then diluted and plated on MSM agar containing 2.5 mM sodium arsenate and 0.004% yeast extract.

All of the isolated strains were routinely grown in lysogeny broth (LB) medium [15] or in MSM medium [NaCl 1.17 (g/L), KCl 0.30 (g/L), NH_4_Cl 0.15 (g/L), MgCl_2_·6H_2_O 0.41 (g/L), CaCl_2_ 0.11 (g/L), KH_2_PO_4_ 0.20 (g/L), Na_2_SO_4_ 0.07 (g/L), and NaHCO_3_ 2.00 (g/L), pH 8.0] supplemented with yeast extract (0.04% w/v) at 22°C. For siderophores production, bacterial strains were cultivated at 22°C in GASN medium [2 g/L L-asparagine, 7 g/L glucose, 0.96 g/L Na_2_HPO_4_, 0.44 g/L KH_2_PO_4_, and 0.2 g/L MgSO_4_·7H_2_O, pH 7.0] [16].

### 2.2. Copper Ore Concentrate and Middlings

Copper ore concentrate and middlings (intermediate during ore processing) were received from KGHM S.A. “Polska Miedź” (Poland). Copper ore concentrate contained 185970 mg Cu/kg, 22169 mg Fe/kg, 2460 mg Pb/kg, 3765 mg As/kg, 533, 1 mg Ni/kg, and 1273 mg Co/kg. Middlings contained 21495 mg Cu/kg, 4290 mg Fe/kg, 291, 5 mg As/kg, 356, 5 mg Ni/kg, and 594 mg Co/kg. The main arsenic minerals are enargite (AsV), tennantite (AsIII), and realgar (AsIII). Arsenopyrite is present as trace mineral. Sandstone, dolomite, and limestone are gangue. Sulphur content is 0.3–0.9% (mainly as sulfides and sulfates).

### 2.3. Arsenopyrite

Crystals of arsenopyrite were received from ancient gold mine in Zloty Stok.

### 2.4. Arsenic Respiration Screening Test

The ability of bacterial isolates to reduce As (V) in respiratory processes was tested using MSM agar plate containing 5 mM sodium lactate as the carbon source and 5 mM sodium arsenate as the electron acceptor. Cultures were grown under anaerobic conditions and, after 7 days of cultivation at 22°C, the agar plates were flooded with 0.1 M AgNO_3_ solution. The reaction between AgNO_3_ and As (III) or As (V) results in the formation of a coloured precipitate [17]. A brownish precipitate reveals the presence of Ag_3_AsO_4_ (silver arsenate) in the medium, while a yellow precipitate shows the presence of Ag_3_AsO_3_ (silver arsenite) (colonies expressing arsenate reductase).

### 2.5. Chemical Analyses

Solid samples (bacterial biomass, powders of copper ore concentrate, and middlings) were thoroughly dried at 60°C, and then 9 mL 65% HNO_3_ and 1 mL 36% H_2_O_2_ were added to 0.25–0.30 g of the dry mass and digested in a closed system with heating in a microwave oven (Milestone Ethos Plus with Lab Terminal 800 Controller, Italy) [18]. Liquid samples (culture supernatants and siderophores solutions) were placed in 12 mL glass vials (Agillent, USA) and mixed with 65% HNO_3_ in a ratio 4 : 1. Quantitative analysis of As, Cu, and Fe was performed by flame atomic absorption spectrometry (FAAS) and graphite furnace atomic absorption spectrometry (GFAAS) (AA Solaar M6 Spectrometer, TJA Solutions, UK) using standard solution (Merck, Darmstadt, Germany) prepared in 0.5 M HNO_3_. Arsenic speciation in culture supernatants was determined as described by Drewniak et al. (2008) [19].

### 2.6. PCR Amplification and Sequencing: 16S rRNA Gene, *arrA* and *arsC* Genes

Amplification of the 16S rRNA genes and the *arsC *genes was performed as described by Drewniak et al. (2008) [20]. For the amplification of the *arrA* genes, primer pair ArrAfwd and ArrArev were used as described by Malasarn et al. (2004) [21]. The PCR products were ligated with the vector pGEM-T-Easy (Promega) and were transformed to chemically competent *Escherichia coli *TG1 cells. Plasmid inserts were sequenced on an ABI3730 DNA analyser (Applied Biosystems) at the Laboratory of DNA Sequencing and Oligonucleotide Synthesis, IBB PAS, using universal M13F and M13R primers. Additional primers 518F and 519R [22] were used for sequencing of the 16S rRNA genes. Partial sequences were assembled using Clone Manager Professional Suite software (version 8) and were verified manually.

### 2.7. Phylogenetic Analysis

The near full-length 16S rRNA gene sequences (**∼**1.4 kbp) from the isolates and reference sequences from the known arsenic-utilizing bacteria were aligned using ClustalW software (http://www.ebi.ac.uk/clustalw). The obtained alignment was adjusted manually and then used to construct a phylogenetic tree. The unrooted tree was constructed using the distance matrix “Neighbor-Joining Method” with NEIGHBOR from the PHYLIP 3.6 software package [23]. Distance matrices were calculated by DNADIST (PHYLIP) using Jukes-Cantor formula. Bootstrap analysis was carried out 1000 times using SEQBOOT (PHYLIP). A consensus tree was computed with CONSENS (PHYLIP).

### 2.8. Determination of the Minimum Inhibitory Concentrations of Metals/Metalloids

To examine the minimal inhibitory concentrations (MIC) of heavy metals, 96-well microplates containing LB medium amended with the respective heavy metal compounds were used. Each well of the microplates was inoculated with cells from fresh overnight cultures to a final density of approximately 10^6^ cells/mL and then incubated for 48 h at 22°C in aerobic conditions. The following metals and their compounds were used for MIC determination: As (III) 0.0–25.0 mM; As (V) 0.0–500 mM; Cu (II) 0.0–5.0 mM; Cd (II) 0.0–5.0 mM and Co (II) 0.0–5.0 mM; and Fe (III) 0.0–25.0 mM, Ni (II) 0.0–5.0 mM, Pb (II) 0.0–20 mM, and Zn (II) 0.0–5.0 mM. The MIC was defined as the lowest concentration of Me^n+^ that completely inhibited bacterial growth.

### 2.9. Detection of Siderophores and Their Chemical Nature

The production of siderophores was examined by aerobic growth experiment on GASN medium [16]. The concentration of siderophores present in culture supernatants was measured using the method described by Schwyn and Neilands [24]. A standard curve was prepared with deferoxamine mesylate (DFOB). The quantity of siderophores produced was determined from the standard curve using CAS assay solution [24] and the absorbance value measured at 630 nm after 1 h of incubation [25] and denoted as mM DFOB. Detection of hydroxamate siderophores was performed using FeCl_3_ test [26] and for catecholates the Arnow test was used [27].

### 2.10. Test for Dissolution of Arsenic Minerals by Siderophores Produced by the Isolates

Modified method described by Drewniak et al. (2010) [10] was used for determination of bacterial siderophores ability to dissolve arsenopyrite (FeAsS) and arsenic minerals (enargite (Cu_3_AsS_4_) and tennantite ((Cu,Ag,Fe,Zn)_12_As_4_S)) contained in copper ore concentrates and middlings. The following modifications were made: in dissolution reactions, 50 mL of 0.2 mM bacterial siderophores and sterile GASN medium (as a control) were added to 0.5 g of a given, sterile mineral, and the concentrations of As and Fe in the solution were measured after 48 h of incubation in aerobic conditions at 22°C using graphite furnace atomic absorption spectrometry (GFAAS) (AA Solaar M6 Spectrometer, TJA Solutions, UK). The method of siderophores preparation was as follows: (i) strains were grown in GASN medium for 24 h at room temperature in aerobic conditions, (ii) the cultures were then centrifuged (10,000 ×g, 10 min.), and the supernatants were used as siderophore preparations. Siderophores concentration was extrapolated from a standard curve and represented in mM of desferrioxamine B (DFOB).

### 2.11. Arsenic Minerals Utilization in Respiratory Processes

The ability of the isolates to utilize arsenic containing minerals (copper ore concentrates and middlings obtained from KGHM S.A, Poland) in respiratory processes was examined in the MSM medium supplemented with 5 mM sodium lactate as the electron donor and as the sole carbon source. Sterilized, powdered samples of minerals were added to the MSM medium (100 mL) to a final concentration of 1% (w/v) and were inoculated with cells from fresh overnight cultures to a final density of approximately 10^6^ cells/mL. The cultures were incubated at 22°C for 21 days under anaerobic conditions, when the ability to use the investigated minerals as a final electron acceptor was tested. Noninoculated samples were used as controls. The cultures were sampled for chemical analyses (pH, heavy metal concentration, and arsenic speciation) and estimation of colony forming unit (cfu) at the start of the experiment and every 7 days.

### 2.12. Nucleotide Sequence Accession Number

The 16S rRNA gene sequence of isolated strains has been deposited in GenBank under accession numbers KF986639, KF986640, KF986641, and KF986642.

## 3. Results and Discussion

### 3.1. Isolation and Identification of Dissimilatory Arsenate Reducers

Microbial mats form sediments sampled from the ancient Zloty Stok gold mine (SW Poland) were used as the source of arsenic-metabolizing microbes, for several reasons. Our previous studies [20] showed that the physical and chemical conditions prevailing in the Zloty Stok mine promote the growth and development of arsenic-transforming microbes. Arsenic in the mine is present in soluble form as arsenite and arsenate in mine waters and also occurs as primary (arsenopyrite and lollingite) and secondary minerals (such as scorodite) deposited in the rocks and sediments [28]. The mine waters and sediments also contain high amounts of other heavy metals (e.g., Cu, Co, Mn, and Zn) and therefore the microbial mats seemed to be an ideal source for the isolation of model dissimilatory arsenate reducing bacteria (DARB) that can be capable of dissolution of arsenic-bearing minerals. Regardless of the mechanism of arsenic release from polymetallic ores/minerals, microorganisms that are involved in dissolution processes should be adequately adapted to the surrounding conditions, for example, resistance to arsenic and heavy metals.

**Figure 1:**
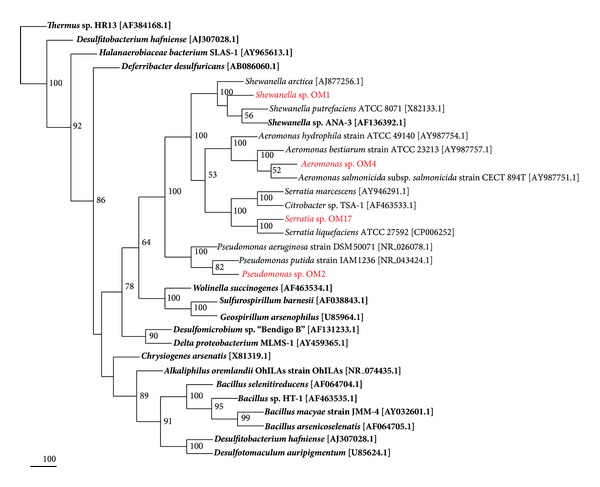
Neighbour-joining tree, computed based on the alignment of 16S rRNA gene sequences, showing the phylogenetic relationship of arsenic-respiring isolates with known arsenic resistant and dissimilatory arsenate reducing bacteria. The analysis included the data from *∼*1400 nucleotide positions. The tree is a consensus of 100 neighbor-joining trees. Percentage values on each branch represent the corresponding bootstrap probability values obtained in 100 replications and numbers are shown only for values of >50%. Sequences derived from arsenic-respiring isolates (OM1, OM2, OM4, and OM17) are indicated in large bold red type; sequences derived from other dissimilatory arsenate reducing bacteria are indicated in black bold type. The remaining sequences indicated in normal type derived from strains that are only resistant to arsenic.

Microbial mats were inoculated into the modified MSM medium supplemented with 2.5 mM sodium arsenate and 5 mM sodium lactate. After 7 days of incubation under anaerobic conditions, enrichments were subcultured twice for 7 days each and the cultures were plated on MSM agar. Morphological observations (colony colours and cell shape), physiological test (AgNO_3_ test), and 16S rRNA gene analysis (ARDRA analysis and sequencing) allowed for the identification of four different strains (OM1, OM2, OM4, and OM17) capable of anaerobic growth with As (V) reduction. BLASTN analysis of partial 16S rDNA sequences (**∼**1.4 kbp) showed that all the isolates belonged to the class *γ*-Proteobacteria. The nearest known phylogenetic relative of the strain OM1, with a sequence similarity of 99%, is *Shewanella* sp. OTUC2. The strain OM2 showed the highest similarity (99%) to genus *Pseudomonas koreensis *strain JH18. The strain OM4 was closely related (100% of similarity) to genus *Aeromonas hydrophila*, whereas strain OM17 was closely related (99% of similarity) to *Serratia liquefaciens* ATCC 27592. Phylogenetic studies confirmed BLASTN analysis and showed that 16S rRNA gene sequences of all isolates are located in clusters of closely related species within the class *γ*-Proteobacteria (Figure 1). Interestingly, two strains, *Aeromonas* sp. OM4 and *Serratia* sp. OM17, are the first described representatives of their genera, which are capable of dissimilatory arsenate reduction. So far described *Aeromonas *spp. and *Serratia *spp. strains were able to reduce arsenate, but only using cytoplasmic reductase as a resistance mechanism [20, 29, 30].

All of the tested bacteria showed a broad range of temperature tolerance: 4–37°C, with the optimum at 30°C (OM1, OM2, and OM17) and 37°C (OM4) (Table 1). The pH optimum was found to be slightly acidic or close to neutral (4–7), but growth at alkaline pH (10) was also observed (Table 1). Ability to grow in a broad range of pH and temperature conditions makes the isolated strains potential biotechnological tools for bioremediation of arsenic-contaminated sites. One of the main factors limiting the use of bacteria in bioremediation technologies is a sensitivity of strains to changing physicochemical conditions. The broad spectrum of tolerance to pH and temperature increases the chances of survival in the environment.

### 3.2. Detoxification Mechanisms

The primary role of microorganisms living under unfavourable environmental conditions is to survive. Detoxification mechanisms that protect against harmful substances are very often connected with processes that require energy supply and sometimes are associated with respiratory processes in which energy is generated. The most common mechanism of detoxification is based on blocking the membrane channels through which toxic substances enter the cell. Another method of protection relies on the active removal out of the cell using specific membrane pumps [31]. Arsenite ions are directly removed by membrane permeases, while arsenates ions require transformation into the arsenites prior to removal. In this study we have verified the level of arsenic resistance and we investigated the presence of cytoplasmic arsenate reductase genes (*arsC*) in the genomes of the isolated strains.

The isolates were hypertolerant to arsenate (250–400 mM) and showed high resistance to arsenite (10–16 mM). *Serratia* sp. OM17 was the most resistant to As (V) isolate, which tolerated concentrations of arsenate up to 400 mM. The highest MIC for As (III) was found for *Aeromonas* sp. OM4, which tolerated concentrations of arsenite up to 16 mM. Such high resistance to arsenic was also found in other arsenic-tolerating microorganisms, but any described arsenic hypertolerant strain was able to use As (V) in dissimilatory arsenate reduction [20, 32]. The presence of *arsC* gene was confirmed in the genomes of all of the tested strains (Table 1). BLASTN results showed that *arsC* genes of *Shewanella *sp. OM1,* Pseudomonas *sp. OM2, and *Serratia *sp. OM17 have a high similarity to cytoplasmic arsenate reductases genes of closely related species. Nucleotide sequence of *arsC *gene of *Shewanella *sp. OM1 is 78% identical to the arsenate reductase gene of *Shewanella putrefaciens* CN-32. The *arsC* sequence of *Pseudomonas *sp. OM2 is 84% identical to the arsenate reductase gene of *Pseudomonas denitrificans* ATCC 13867, while the *arsC* of *Serratia *sp. OM17 is 96% identical to the arsenate reductase gene of *Serratia liquefaciens* ATCC 27592. In turn, the *arsC* gene of *Aeromonas *sp. OM4 is 76% identical to arsenate reductase of *Acidovorax ebreus* TPSY. Phylogenetic analysis showed that the *arsC *genes of the OM isolates are closely related to the arsenate reductase gene sequences of known arsenic resistant strains, but not necessarily from the same species (see Figure S1 available online at http://dx.doi.org/10.1155/2014/841892).

In the context of arsenic minerals dissolution, equally important as arsenic resistance is the tolerance to other heavy metals, such as Cu, Co, Cd, Zn, Fe, Ni, and Pb. The level of resistance to the tested metals was between 0.25 and 4 mM: Cu (II) (for all strains up to 4 mM), Co (II) (OM1 and OM17 0.75 mM, OM2 0.5 mM, and OM4 1 mM), Cd (II) (OM1, OM2, and OM4 0.25 mM and OM17 0.5 mM), Zn (II) (OM1 and OM 17 up to 3.5 mM, OM2 1 mM, and OM4 0.75 mM), Fe (III) (for all the strains up to 2 mM), Ni (III) (OM1 and OM4 up to 3 mM and OM2 and OM17 up to 2.5 mM), and Pb (II) (for all the strains up to 2 mM). Such heavy metal multiresistance enables their active growth in environments rich in heavy metals.

### 3.3. Description of the Process That Promotes Arsenic Mobilization

The mechanisms of microbial arsenic mobilization depend on many biogeochemical factors, among which the most important seem to be the following: the presence of the appropriate physiological group of microbes, type of minerals (primary or secondary), pH (acidic or neutral), Eh (reductive or oxidative conditions), and the availability of oxygen or other electron acceptors. Direct mechanisms of arsenic mobilization include oxidative dissolution of primary arsenic minerals and reductive dissolution of secondary arsenic minerals [6].

#### 3.3.1. Arsenate Respiration Process

**Figure 2:**
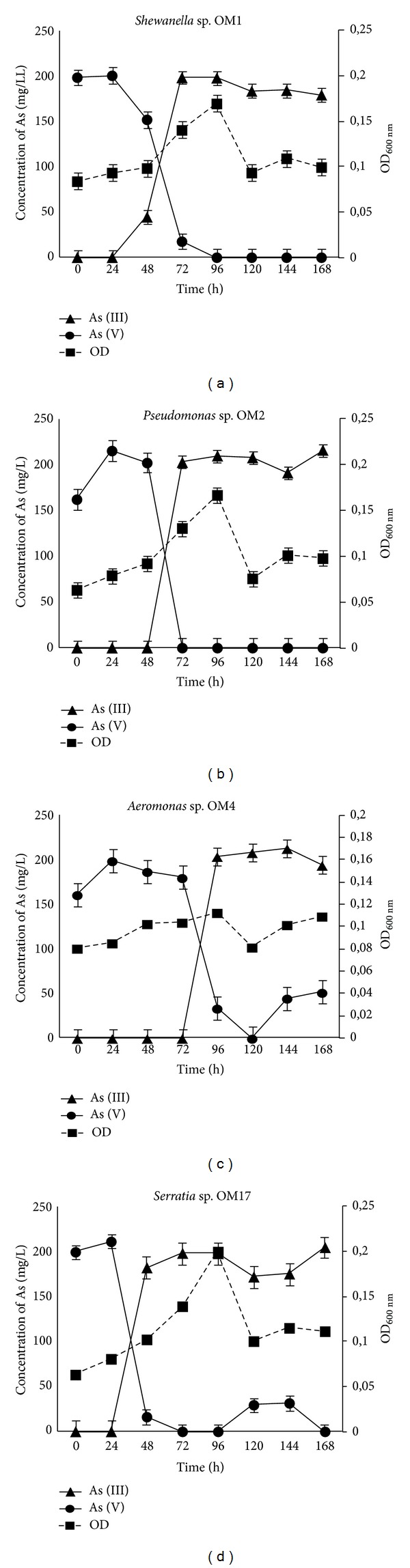
Dissimilatory arsenate reduction mediated by *Shewanella* sp. OM1, *Pseudomonas* sp. OM2, *Aeromonas* sp. OM4, and *Serratia* sp. OM17. Arsenate respiratory processes were tested using cultures grown under anaerobic conditions in the MSM medium, supplemented with 2.5 mM sodium arsenate and 5 mM sodium lactate.

To test the isolates for the ability to grow by arsenate respiration, anaerobic growth experiments were conducted. All of the isolates grew with 5 mM sodium lactate as the electron donor and 2.5 mM sodium arsenate as the electron acceptor (Figure 2). In almost all of the cultures (except *Aeromonas* sp. OM4) arsenate reduction was observed during the exponential phase, in which the growth rate and the rate of As (V) reduction were proportional. Growth did not occur when medium without arsenate was used or arsenate was completely utilized, indicating that arsenate was required for growth. The *Serratia *sp. OM17 strain was found to be the most efficient isolate, in terms of the highest arsenate reduction rate [7.81 mg As (V) ·L^−1^·h^−1^]. Complete reduction of 2.5 mM (187.5 mg·L^−1^) arsenate into arsenite was observed after 48 h of incubation. Other strains had a slightly lower arsenate reduction rate, and the complete transformation of As (V) into As (III) required at least 72 hours of incubation.* Shewanella *sp. OM1 and *Pseudomonas* sp. OM2 completely reduced 2.5 mM of As (V) to As (III) within 72 hours, while *Aeromonas *sp. OM4 within 96 hours. In addition to the lowest As (V) reduction rate, *Aeromonas *sp. OM4 was not able to completely reduce arsenates.

All of the tested strains were unable to reduce the arsenate at concentrations above 2.5 mM, despite their ability to tolerate high concentrations of As (III) (10–16 mM) and As (V) (250–400 mM). Interestingly, most of dissimilatory arsenate reducers isolated during similar studies were able to reduce arsenate at much higher concentrations, exceeding 10 mM [11, 33] and sometimes reaching up to 40 mM [34], although they come from environments in which the arsenic is present at a similar level as in Zloty Stok gold mine (isolation site of the tested strains).

Similar to other known arsenic respiratory strains, the *arrA* gene (coding for the large subunit of dissimilatory arsenate reductase) was found in the genomes of all of the isolates. BLASTN results showed that all dissimilatory arsenate reductase genes of the isolates (OM1, OM2, OM4, and OM17) are closely related to *arrA* genes detected in metagenomic studies and described as uncultured bacterium clones. In turn, phylogenetic studies with the use of *arrA *sequences of the isolates and known arsenic respiratory bacteria showed that arsenic respiratory genes of the OM (1, 2, 4, and 17) isolates form a common, separate cluster and they are not closely related to the genes of their phylogenetic relatives.

#### 3.3.2. Dissolution of Arsenic Containing Minerals by Metabolites Produced by DARB

Nutrient uptake from minerals may be performed by different mechanisms. Microorganisms may cause disaggregation of minerals through colonization and physical penetration of the mineral surface [35]. Extraction of vital elements from the crystal lattice of minerals may be also supported by organic agents produced by the cells. Among the most common microbial organic agents involved in minerals dissolution are siderophores: high-affinity, metal-binding compounds secreted outside the cell envelope that can chelate metal ions and bind to atoms on the mineral surface. They can effectively bind many metal cations including Fe, Mg, Mn, Cr, Ga, Pl, Pb, Cd, Zn, Cu, Ni, U, Co, Sn, and As [36–40].

Biochemical tests showed that under iron-limiting conditions all the DARB isolates produce siderophores that are classified to the hydroxamate-type. The highest concentration of siderophores in GASN medium after 48 h of incubation at 22°C was noted in the culture of *Aeromonas* sp. OM4 (118 *μ*M) and the lowest in the *Shewanella *sp. OM1 culture (88 *μ*M). *Pseudomonas *sp. OM2 and *Serratia *sp. OM17 produced siderophores at similar levels, 116 *μ*M and 110 *μ*M, respectively.

A further issue that needs clarification is the ability of the siderophores, produced by the selected strains, to dissolve (i) arsenopyrite and (ii) middlings and copper concentrates containing arsenic minerals (enargite, realgar, and tennantite). For this purpose, cell-free metabolites (containing siderophores) obtained from the cultivation of the DARB isolates under iron and arsenic limiting conditions in GASN medium were used.

**Figure 3:**
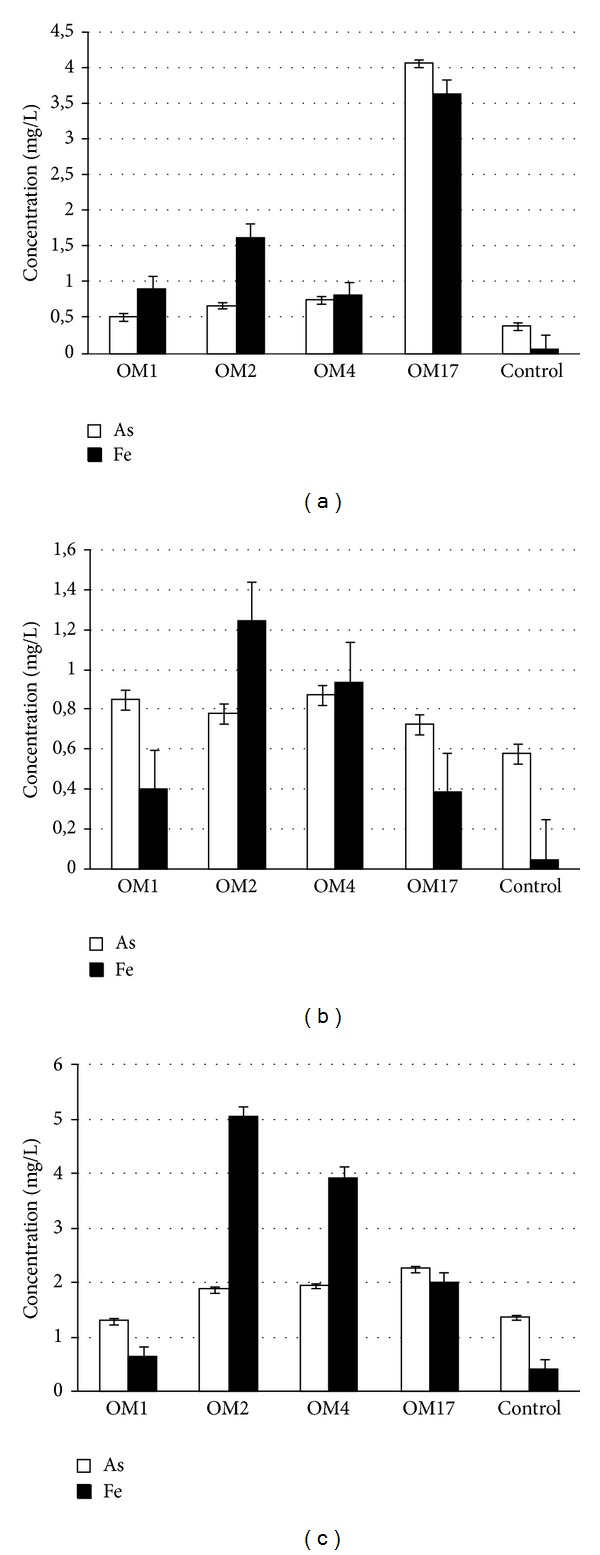
Release of arsenic and iron from arsenopyrite (a), middlings (b), and copper ore concentrates (c), after 48 h of incubation with sterile GASN medium as a control, and siderophores produced by the isolated strains: *Shewanella* sp. OM1, *Pseudomonas* sp. OM2, *Aeromonas* sp. OM4, and *Serratia* sp. OM17.

After 48 h of leaching of arsenic minerals by the DARB siderophores, iron and arsenic were released into the solutions of all of the tested samples. Siderophores produced by most of the tested strains show the capacity to release arsenic from minerals at a level similar to the control. In the case of arsenopyrite dissolution by siderophores, only metabolites produced by *Serratia* sp. OM17 showed significant differences in the release of arsenic in relation to the control sample. The concentration of arsenic in the OM17 siderophores solution was almost eleven times higher than in the control sample (4.07 mg/L of As was noted in *Serratia* sp. OM17 siderophores solution, whereas 0.37 mg/L in sterile GASN medium). Twice as high concentration of arsenic than in the control was observed in the siderophores solution of the *Aeromonas* sp. OM4 strain. The concentration of arsenic mobilized from arsenopyrite by siderophores produced by two other strains (OM1 and OM2) was slightly higher (1.37 and 1.8 times, resp.) than in the control (Figure 3(a)). The release of arsenic from copper concentrate and middlings by siderophores was inefficient and arsenic concentrations were at the level of the control. The highest concentration of arsenic in the middlings dissolution was observed when the siderophores solution of *Shewanella *sp. OM1 was used (1.47 times higher than the control) (Figure 3(b)). In the case of copper ore concentrates, the highest concentration of arsenic was observed when siderophores solution of *Serratia *sp. OM17 was used (1.66 times higher than in the control) (Figure 3(c)).

In contrast to arsenic case, the iron concentration in almost all of the samples was at a much higher level than in the control. The highest iron concentration in arsenopyrite sample was observed in the siderophores solution of* Serratia* sp. OM17 (Figure 3(a)), in which the concentration of Fe exceeded ~72 times the iron content recorded in the control sample. In the middlings and copper ore concentrates samples, the highest iron concentrations were observed when the siderophores solution of *Pseudomonas *sp. OM2 was used (25 times and 12.5 higher than the control in the middlings and copper concentrates, resp.) (Figures 3(b) and 3(c)). Regardless of the copper mineral, the lowest concentration of iron was noted in the case of siderophores produced by *Shewanella *sp. OM1 (only 1.5 times more than in the control with copper concentrates) (Figure 3(c)).

The obtained results demonstrate that siderophores produced by dissimilatory arsenate reducers are involved in dissolution of arsenic containing minerals. Based on the results it can be concluded that the mechanism of arsenic minerals dissolution can be driven by iron uptake through siderophores, while arsenic is released simultaneously as by-product. These results are consistent with the previously described arsenic mobilization mechanisms used by dissimilatory iron reducers [14, 41].

### 3.4. Removal of Arsenic from As-Containing Minerals

It is commonly known that the release of arsenic from As-bearing minerals may take place by two main processes: (i) oxidation of the primary arsenic minerals and (ii) reductive dissolution of the secondary minerals. Primary As-minerals such as arsenopyrite or realgar can be dissolved by direct or indirect mechanisms in the presence of oxygen by chemolithoautotrophic microorganisms [42]. In turn, under reducing conditions, microorganisms can use arsenate adsorbed on the surface of iron minerals (e.g., scorodite or ferrihydrite) as a terminal electron acceptor in arsenic respiration [14, 43].

In the literature, there is not much information concerning the release of arsenic from the primary minerals under reducing conditions. Thus, it is interesting to investigate if under anaerobic conditions bacteria are capable of arsenic mobilization from primary minerals and in particular whether they are able to use such minerals in the process of respiration as an electron donor. In order to verify the above hypothesis we performed respiration experiments using primary arsenic minerals as the final electron acceptors. Arsenopyrite (FeAsS), as one of the most common primary arsenic minerals, containing arsenic in reduced form, was used in this experiment as a control in which bacterial cells have not final electron acceptor. Copper concentrates and middlings were used as a source of primary arsenic mineral (such as enargite), in which arsenic occurs as arsenate. Both Cu-sources are polymetallic and contain arsenic as impurities (0.37% for the concentrates and 0.029% for the middlings). The ability of the isolates to utilize arsenic containing minerals in respiratory processes was examined in the MSM medium supplemented with 5 mM lactate as the electron donor and the sole carbon source and 1% of appropriate mineral as a source of electron donors.

**Figure 4:**
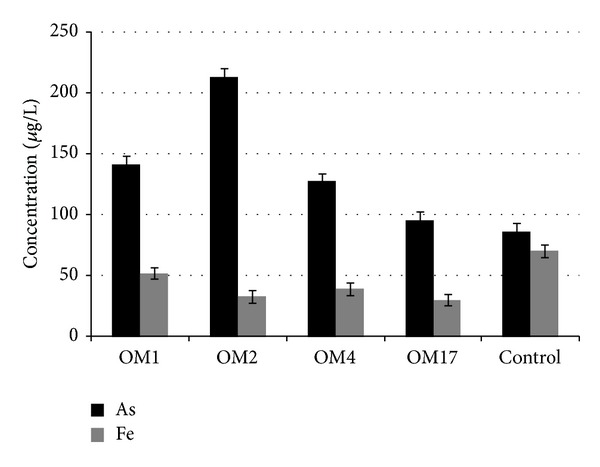
Arsenopyrite dissolution under anaerobic conditions by dissimilatory arsenate reducing bacteria: *Shewanella* sp. OM1, *Pseudomonas* sp. OM2, *Aeromonas* sp. OM4, and *Serratia* sp. OM17. As and Fe concentrations in culture liquid after 21 days of incubation under anaerobic condition in MSM medium supplemented with 1% FeAsS and 5 mM sodium lactate.

As predicted, none of the isolated strains were capable of using arsenopyrite as a respiratory substrate. Growth was not observed in any culture, but only death phase. Number of colony forming units decreased from the initial 10^6^ cfu/mL to 10^2^–10^3^ cfu/mL after 21 days of incubation in all cultures. There were no significant changes in pH. After 7 days of incubation pH in all cultures increased to ~8.5 and remained stable until the end of the experiment (in control sample the pH was stable throughout the experiment). The concentrations of arsenic and iron in the supernatants exceeded the levels observed in the control sample (sterile medium), but the amount of mobilized arsenic and iron was low (Figure 4), especially if the total arsenic and iron content in arsenopyrite was taken into account. The mobilization of small amounts of As and Fe from FeAsS by the cells being in decline phase can be explained by an unspecific dissolution of arsenopyrite by metabolites (e.g., ligands and organic acids) released during cell lysis. Thus dissolution in reductive conditions mediated by dissimilatory arsenate reducing bacteria is not the main driving force leading to arsenic mobilization from arsenopyrite, but only a passive process that should be taken into account during long-term environmental risk assessment. These results are in accordance with the current knowledge about the microbiological dissolution of the primary arsenic minerals; that is, the primary arsenic minerals are mainly utilized under aerobic conditions by oxidative dissolution [6].

**Figure 5:**
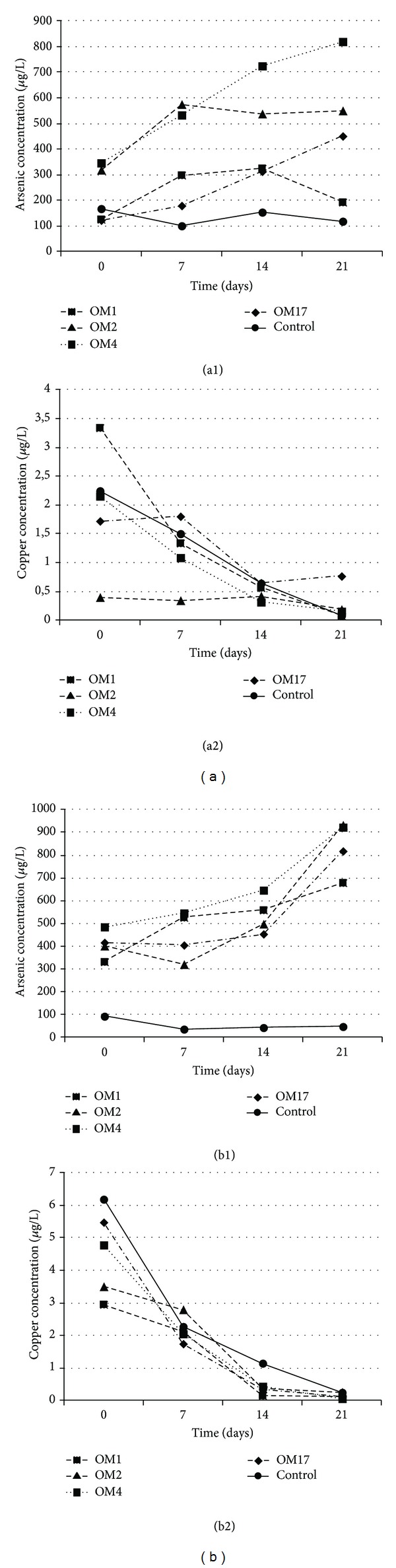
Dissolution of As-bearing copper minerals mediated by dissimilatory arsenate reducing bacteria. Arsenic (1) and copper (2) release during 21 days of incubation in MSM medium supplemented with 5 mM sodium lactate and (a) 1% middlings and (b) 1% copper ore concentrates.

**Figure 6:**
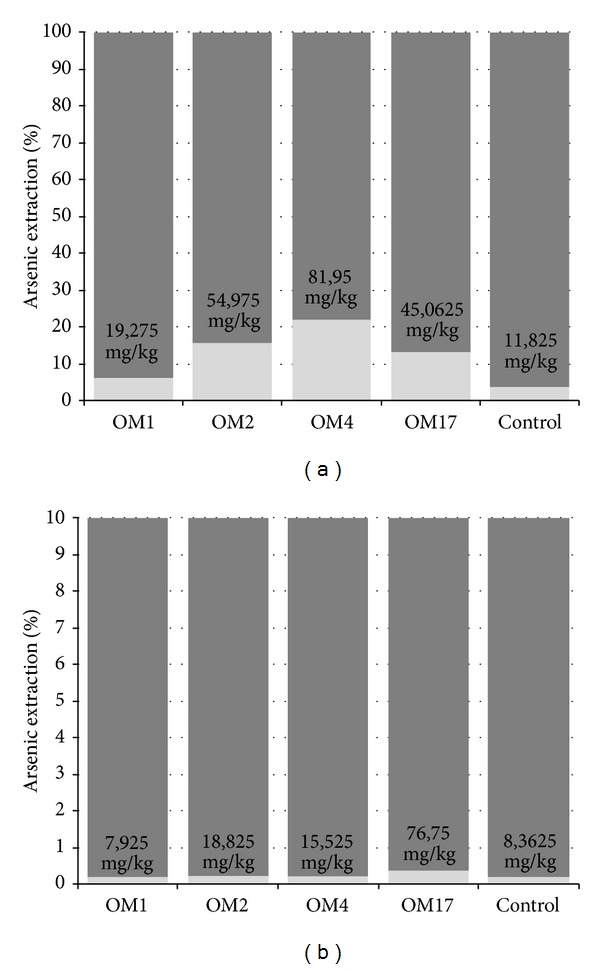
Comparison of effectiveness of arsenic removal from middlings (a) and copper ore concentrates (b) mediated by dissimilatory arsenate reducing bacteria: *Shewanella* sp. OM1, *Pseudomonas* sp. OM2, *Aeromonas* sp. OM4, and *Serratia* sp. OM17.

A quite different effect was observed in the respiration experiments with arsenic containing copper sulfide minerals used as the final electron acceptor. All of the strains were capable of growing under anaerobic conditions in the MSM medium containing (1%) sterile Cu-minerals as the sole electron acceptor and considerable arsenic concentrations were detected in all of the culture supernatants after 21 days of incubation (Figure 5). In almost all of the cultures, the concentration of arsenic in the culture supernatants increased in time (Figures 5(a1) and 5(b1)) and correlated with the rates of growth (data not shown). The final amount of the removed arsenic oscillated between 19.28 and 81.95 mg/kg (6.6% and 28.1%) for the middlings (Figure 5(a1)) and 7.93 and 76.75 mg/kg (0.21% and 2.03%) for copper ore concentrates (Figure 5(b1)). The most efficient strain, in terms of arsenic release, was *Aeromonas *sp. OM4 strain cultured in middlings and copper ore concentrate (As concentration in culture liquid reached 819.50 and 922.50 *μ*g/L, resp.). The lowest level of arsenic removal was observed for *Shewanella *sp. OM1 culture (As concentration in culture liquid was 192.75 *μ*g/L for middlings and 678.75 *μ*g/L for copper ore concentrates) (Figures 5(a1) and 5(b1)). Irrespective of which strain was used, the effectiveness of arsenic extraction was 10 times higher in the middlings than in the copper ore concentrates leaching experiment. The maximum recovery of arsenic from middlings was 28.11% after 21 days for *Aeromonas *sp. OM4 (Figure 6), while the maximum recovery of arsenic from copper ore concentrates was 2.47% at 21 days for *Pseudomonas* sp. OM2 (Figure 6). For all of the strains, the arsenic recovery process was selective and was not associated with simultaneous removal of copper (Figures 5(a2) and 5(b2)). High concentration of copper was noted in all of the samples at the beginning of cultivation (Figures 5(a2) and 5(b2)), which was probably associated with chemical leaching of copper (by medium components and residual metabolites from the overnight cultures). Moreover, in all of the cultures and control samples, copper concentration decreased with time. Only in the case of *Serratia *sp. OM17 cultured on middlings, elevated level of copper leaching (767.50 *μ*g/kg) was reported after 21 days of incubation. However, it should be noted that the resulting value represents only 0.04% of the initial copper content. These results may indicate the slow copper precipitation process under neutral conditions (pH fluctuated around 7.5 ± 0.4 during the experiment) and absence of microbial activity connected with the leaching of copper.

On the basis of the above results we can postulate that arsenic can be released from some As-bearing polymetallic minerals (such as copper ore concentrates or middlings) in reductive conditions by dissimilatory arsenate reducers. However, the mechanisms of arsenic release are still unknown. Comparing the data from the arsenopyrite and copper concentrates/middlings respiration experiment, we can only assume that the mechanism of arsenic release from Cu-minerals is indirect. To understand how the DARPs release arsenic from minerals, deep physiological and microbe-mineral interaction (including arsenic speciation) analysis is needed. The ability to use different inorganic electron donors (e.g., Fe^2+^ or S^2-^), which may be conjugated to electron acceptors other than oxygen (e.g., NO_3_^−^  and As_3_O_5_^3−^), requires verification. Similarly, the hypothesis that bacteria can utilize polymetallic sulfide as a source of enzyme cofactors such as Mo, Cu, Zn, and Mg [35] must be also verified.

## 4. Conclusions

In this work, we have shown that dissimilatory arsenate reducing bacteria have a great potential for arsenic mobilization into the environment, not only from secondary arsenic minerals but also from the primary arsenic minerals deposited in polymetallic ores. All of the isolated strains give positive results in the arsenic respiratory test. They effectively grew with sodium lactate as the electron donor and sodium arsenate as the electron acceptor. Resistance to high concentrations of arsenite and arsenate, as well as a number of other heavy metal compounds, was another common feature of the isolates. Moreover, all of the strains displayed the ability to grow in a broad range of pH values and temperatures and under both aerobic and anaerobic conditions. Under iron-limiting conditions all of the isolated DARBs produced siderophores that were involved in dissolution of arsenic containing minerals. None of the isolated strains were capable of using arsenopyrite as a respiratory substrate, but (low efficient) arsenopyrite dissolution in reductive conditions was observed. We postulate that it was only as a passive process, in which metabolites (e.g., ligands and organic acids) released during cell lysis may play a key role. In turn, utilization of As-bearing copper minerals in respiratory processes was efficiently carried out by most of the isolates. The DARBs strains were capable of growing under anaerobic conditions using As-bearing polymetallic minerals as the sole electron acceptor. Arsenic release from Cu-minerals was selective (not associated with simultaneous removal of copper) and as we concluded the process was indirect. This work clearly showed that the release of arsenic from primary As-minerals included in the polymetallic ores or wastes from their processing (such as copper concentrates and middlings) is possible under reducing conditions, but the mechanism is still unknown and requires further, detailed mineral-microbe interaction studies (to complete understanding).

## Supplementary Material

Phylogenetic analysis of the cytoplasmic arsenate reductase gene (arsC) and the respiratory arsenate reductase gene (arrA). Table S1: Nucleotide sequences of cytoplasmic arsenate reductase (arsC) and respiratory arsenate reductase (arrA) genes. Figure S1: Phylogenetic tree of the cytoplasmic arsenate reductase gene (arsC). Figure S2: Phylogenetic tree of the respiratory arsenate reductase gene (arrA).

## Figures and Tables

**Table 1:** Tolerance to arsenic and arsenate reductase activity and the presence of arsenic metabolism genes.

Strain	Temperature (°C)		pH		MIC for AsV (mM)	MIC for AsIII (mM)	Presence of genes coding for cytoplasmic and respiratory arsenate reductases	
	Range	Optimum	Range	Optimum			*arsC *	*arrA *
*Shewanella *sp. OM1	4–37	30	5–10	7	350	10	+	+
*Pseudomonas *sp. OM2	4–37	30	5–9	5	250	14	+	+
*Aeromonas *sp. OM4	4–37	37	5–8	5	350	16	+	+
*Serratia *sp. OM17	4–37	30	4–10	4	400	12	+	+
