# Supplementary material for: Dissolution of Arsenic Minerals Mediated by Dissimilatory Arsenate Reducing Bacteria: Estimation of the Physiological Potential for Arsenic Mobilization
Source: Biomed Res Int. 2014 Mar 2;2014:841892. doi: 10.1155/2014/841892 (PMC3958731; doi:10.1155/2014/841892)
Supplement: Supplementary file 1 — Phylogenetic analysis of the cytoplasmic arsenate reductase gene (arsC) and the respiratory arsenate reductase gene (arrA). Table S1: Nucleotide sequences of cytoplasmic arsenate reductase (arsC) and respiratory arsenate reductase (arrA) genes. Figure S1: Phylogenetic tree of the cytoplasmic arsenate reductase gene (arsC). Figure S2: Phylogenetic tree of the respiratory arsenate reductase gene (arrA). [file 841892.f1.pdf]

Supplementary data

Table S1. Nucleotide sequences of cytoplasmic arsenate reductase (*arsC*) and respiratory arsenate reductase (*arrA*) genes

**Strain**	**Nucleotide sequence 5'→3'**
**cytoplasmic arsenate reductase (*arsC*) genes**	
Shewanella sp. OM1	GTGCTCTTTCTATGTACCCATAATGCCTGTCGCAGCATCCTAGCTGAAGCTATAGGTCGAGATCTCG<br>TCGGTAAGCAAGCTTTGACTACTATTGCTCAATGGCAATTTGCGAGTGCCGGTCGTGAACCTGCCG<br>GGGTCGTTCATCCTCAAACTCTATTGCAACTAGCACACAGAGGCTATGTTACCGAAGGGCTCTCCAG<br>CAAAAGCTGGGTTATGATGGCGGATTTCACTCCTGACTTAGTGATCACTGTTTGTGATAATGCTGCG<br>GGAGAAACCTGTCCTTTGTGGTTAGGTCAAACACTTAAATTACATTGGGGTTTACCCGACCCAACAT<br>CAATCGACGCCCCCGATATAGATGAGCAATTTAGCTATGTTATAGAAACACTT
Pseudomonas sp. OM2	AGATTAGTCCTGTTCATGTGCACGGCCAACAGCTGCCGCAGCATCCTCTCTGAAGCCATGTTCAATC<br>ATCTGGCGCCAGCGGGATTCGAAGCGGTGAGTGCCGGCAGCTTCCCCAAAGGTCAGGTGTTGCCG<br>CGCAGCCTGTCGACGCTGCAACAGGCCGGCATCGCCATTGATGGTTTGCACAGCAAAGGCAACGAC<br>GCTTTTGAAAGCAACCCGCCGGACATCGTCATCACCGTGTGCGACAAAGCCGCTGGCGAGACTTGC<br>CCGGTGTATTTCGGCCCGGCGCTGAAATCCCACTGGGGGCTGGAAGATCCCTCCGAAGTGAAGGG<br>CGACGAAGCCACTGTCGACGCGGCATTCCACGCCAAATCAC
Aeromonas sp. OM4	ATGCACAGGCAACTCTTGCCGTTCCATTCTGGCAGAGGCGACCTTTAACCACCTGGCTCCAGCCGGT<br>TGGCATGCCATGAGTGCCGGTAGTCAGCCAACCGGTGAGGTGCATCCCCGTTCACTGGCGTTATTG<br>ACCCGCGAAGGTATCGATATCAGCGGATTGCACAGCAAATCCTGGCATGAGCTGCCGGTGACGCCC<br>GATGTTGTGATCAGCGTGTGTGGTAACGCGGCCAATGAAACCTGTCCTGTCTATTTGGGGCCTGTAT<br>TGCGAGCCCACTGGGGCGTAGATGATCCCGCCCATGTGACCGGCAGCGAGGAGCAGATAGCGCTC<br>GCCTTTCACTGTGCATACTCGACGCTGAGAAAGCGTATCGAAGCCCTCTTGTCGCAG
Serratia sp. OM17	ACGTACCAGCCGGCTTGCGGCACATCGCGCAATACGTTGGCACTGATCCGCAACAGTGGTGTGGAA<br>CCGACGGTGATTTTGTATCTGGAGACACCGCCCGACCGTCAGCAATTGCTCAAGCTGATTGCCGATA<br>TGGGGATATCTGCGCGAGCCTTATTGCGTAAAAACGTGGAGCCTTATGAATCGTTGGGGCTGGCGG<br>AGGTAGGCTGGAGTGACGAACAGTTGATCGACTTTATGTTGCAACAGCCGATATTGATTAATCGAC<br>CTATTGTGGTGACACCCTTGGGCACCCGTCTGTGTCGACCGTCGGAAGCCGTGCTGGATATCCTGCC<br>GGATCCGCAGCAAGGTCCTTTCACTAAGGAAGACGGTGAAAAAGTA
**respiratory arsenate reductase (*arrA*) genes**	
Shewanella sp. OM1	GCCCTTAAGGTGTATGGAATAAAGCGTTTGTTGGAGATTTTATTGAGGGTAAAAACCTGTTTAAAGC<br>GGGTAAAACCGTCCGTATCGAGAGCTTTAAAGAAAGCCATACCTACGGTTTAGTGGAGTGGTGGAA<br>CCAAGCTCTTAAAGACTATACCCCGAAGTGGGCACCTGAAATCACAGGAAGGGC
Pseudomonas sp. OM2	GCCCTTAAGGTGTATGGAATAAAGCGTTTGTTGCCGATTTCACTGACCCTGACCAGCGCTTTGTCAC<br>CGGGCAGCCGGTGAGCGCAGAGCTCTTCGCCGAGCGTCATACCCATGCTCTGGTGCAATGGTGGA<br>ATCTAGAACTGAAAGATCGGACACCCAAATGGGCACCTGAAATCACAGGAAGGGC
Aeromonas sp. OM4	GATAAGGTGTATGGAATAAAGCGTTTGTGGGAGACTTCACTGACCCTGAGCAGCGCTTTGTCACCG<br>GGCAGCCGGTGAGCGCAGAGCTCTTCGCCGAGCGTCATACCCATGGTCTGGTGCAATGGTGGAAT<br>CTGGAACTGAAAGATCGGACACGCCGAAATGGGCACCTGAAATCACAGGAATCACTAGT
Serratia sp. OM17	AAGGTGTATGGAATAAAGCGTTTGTGGGCGACTTCACCGACCCTGAGCAGCGCTTTGTGGACCGGC<br>CAGCCGGTGAGCGCAGAGCTCTTCACCGAGCGTCATACCCATGGTCTGGTGCAATGGTGGAATCTG<br>GAACTGAAAGATCGGACCCCCAAATGGG CACCTGAAAT CACAGG

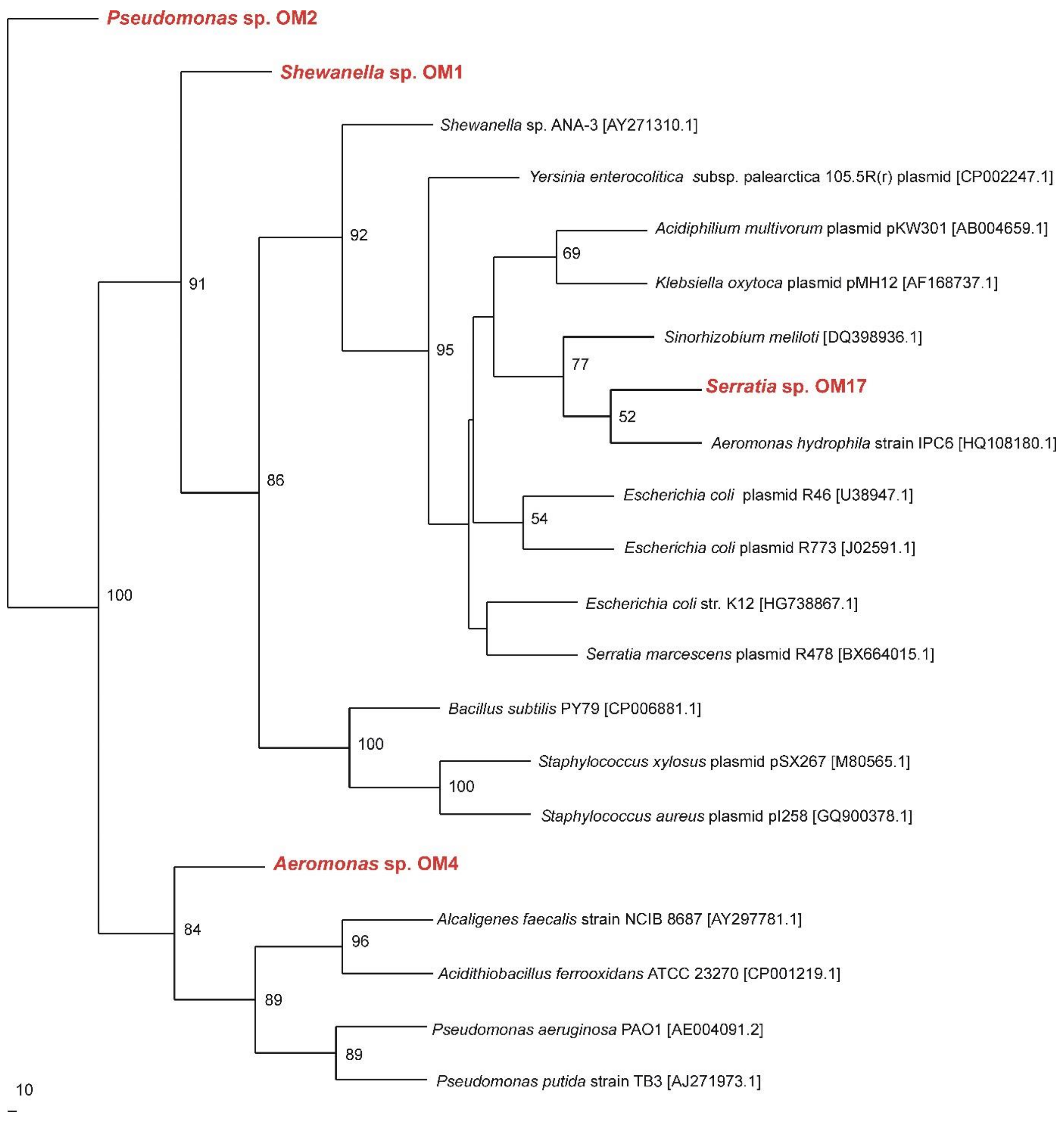

Figure S1. Phylogenetic tree of the cytoplasmic arsenate reductase gene (***arsC***) based on 21 sequences. The unrooted tree was constructed using the Maximum Parsimony algorithm according to Kimura model. The statistical support for the internal nodes was determined by 100 bootstrap replicates. Values of >50% are shown. Accession numbers of the nucleotide sequences used for the phylogenetic analysis are given in parentheses. Sequences derived from arsenate respiratory isolates (OM1, OM2, OM4, OM17) are indicated in large bold red type, sequences derived from others strains are indicated in black type.

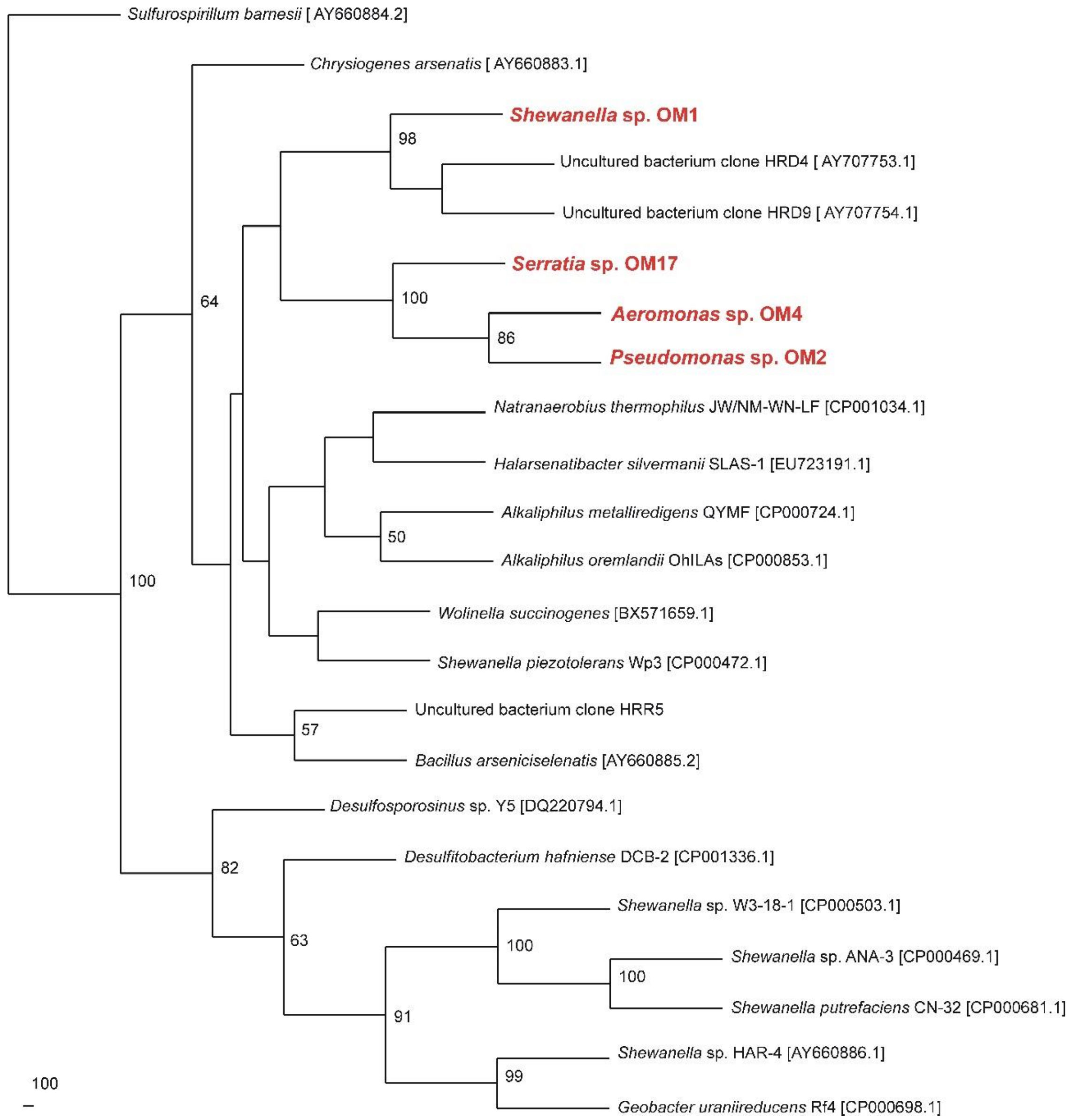

Figure S2. Phylogenetic tree of the respiratory arsenate reductase gene (*arrA*) based on 23 sequences. The unrooted tree was constructed using the Maximum Parsimony algorithm according to Kimura model. The statistical support for the internal nodes was determined by 100 bootstrap replicates. Values of >50% are shown. Accession numbers of the nucleotide sequences used for the phylogenetic analysis are given in parentheses. Sequences derived from arsenate respiratory isolates (OM1, OM2, OM4, OM17) are indicated in large bold red type, sequences derived from others strains are indicated in black type.
